# Neural Mobilization Treatment Decreases Glial Cells and Brain-Derived Neurotrophic Factor Expression in the Central Nervous System in Rats with Neuropathic Pain Induced by CCI in Rats

**DOI:** 10.1155/2017/7429761

**Published:** 2017-03-22

**Authors:** Aline Carolina Giardini, Fabio Martinez dos Santos, Joyce Teixeira da Silva, Mara Evany de Oliveira, Daniel Oliveira Martins, Marucia Chacur

**Affiliations:** ^1^Department of Anatomy, Laboratory of Functional Neuroanatomy of Pain, Institute of Biomedical Sciences, University of São Paulo, São Paulo, SP, Brazil; ^2^University Nove de Julho, São Paulo, SP, Brazil

## Abstract

*Background*. Glial cells are implicated in the development of chronic pain and brain-derived neurotropic factor (BDNF) released from activated microglia contributes to the nociceptive transmission. Neural mobilization (NM) technique is a method clinically effective in reducing pain sensitivity. Here we examined the involvement of glial cells and BDNF expression in the thalamus and midbrain after NM treatment in rats with chronic constriction injury (CCI). CCI was induced and rats were subsequently submitted to 10 sessions of NM, every other day, beginning 14 days after CCI. Thalamus and midbrain were analyzed for glial fibrillary acidic protein (GFAP), microglial cell OX-42, and BDNF using Immunohistochemistry and Western blot assays.* Results*. Thalamus and midbrain of CCI group showed increases in GFAP, OX-42, and BDNF expression compared with control group and, in contrast, showed decreases in GFAP, OX-42, and BDNF after NM when compared with CCI group. The decreased immunoreactivity for GFAP, OX-42, and BDNF in ventral posterolateral nucleus in thalamus and the periaqueductal gray in midbrain was shown by immunohistochemistry.* Conclusions*. These findings may improve the knowledge about the involvement of astrocytes, microglia, and BDNF in the chronic pain and show that NM treatment, which alleviates neuropathic pain, affects glial cells and BDNF expression.

## 1. Introduction

Nerve injury in humans often results in persistent or chronic neuropathic pain which is characterized by spontaneous burning pain accompanied by hyperalgesia and allodynia [1]. The occurrence of lesions in the peripheral nervous system (PNS) and in the spinal cord contributes to the development of neuropathic pain. This pain may be attributed to sensory dysfunction and disability in the peripheral or central nervous system. Studies have shown that chronic pain induced by sciatic nerve injury can be attributed to a combination of factors such as anatomical changes, neurochemistry, and inflammatory expression of ion channels in the central nervous system and other factors [2, 3]. Sciatic nerve lesions not only induce peripheral changes but also induce cortical and subcortical changes in the central nervous system (CNS) [4]. The midbrain periaqueductal gray (PAG) is an important supraspinal pain-regulating center [5], and the thalamic nuclei are pivotally involved in the descending modulation of noxious mechanically and heat evoked responses [6]. However, the mechanisms of nociceptive modulation are still unclear. In this study, we examined the ventral posterolateral and centromedial nucleus of the thalamus and PAG of the midbrain.

Many studies have demonstrated the involvement of glial cells [7] in both peripheral and central injury-induced pain [8–10]. But the importance of glial cells in spinal nociceptive processes was first demonstrated by Garrison et al. (1991), who showed that the density of glia, specifically astrocytes, in the spinal cord increased after sciatic nerve ligature [11]. Further, the thermal hyperalgesia due to subcutaneous injection of formalin or the intraperitoneal injection of endotoxin is blocked by intrathecal injection of glial inhibitors [12]. Glial cells synthesize a large number of substances, many of which are also released by nociceptive neurons that modulate nociceptive responses; among these substances are prostaglandins, glutamate, arachidonic acid, nitric oxide (NO), cytokines, and BDNF [13, 14]. A study shows that the additional BDNF release by activated microglia increases BDNF/TrKB signaling, inducing additional microglial activation. This positive feedback results in a prolonged microglial activation which may enhance the development of neuropathic pain [15]. The inhibition of endogenous BDNF or its receptor TrkB has been found to reduce pain in most experimental studies. Further, exogenous administration of BDNF induces thermal hyperalgesia and mechanical allodynia, and intrathecal injection of anti-BDNF antibody reduces thermal hyperalgesia [16].

The NM technique is a noninvasive manual therapy method used by physiotherapists to treat patients with pain of neural origin, such as the pain caused by compression of the sciatic nerve. Previous studies from our group have demonstrated the efficacy of this technique in relieving induced neuropathic pain in rats [17]. The technique aims to restore the mobility and elasticity of the peripheral nervous system that are lost due to strains that are imposed on the nerve trunks, roots, nerves, spinal cord, nerve wrappings, and the meninges by oscillations and joint angles. The growth of various physiotherapy techniques involving manual therapy that have been studied by researchers (e.g., global postural reeducation [18], joint mobilization [19–21], and the NM technique) has gradually resulted in the reaping of the fruits of new knowledge [22–24].

As our group has previously demonstrated the NM technique's efficacy in relieving pain-related behaviors, our goal here was to evaluate the central involvement of glial cells and brain-derived neurotrophic factor in the thalamus and midbrain of neuropathic rats that had received NM therapy.

## 2. Methods

### 2.1. Animals

Male Wistar rats weighing between 200 and 220 g (2 months old) were used in all experiments. The rats were housed five per cage and maintained on a 12 : 12 h light/dark cycle. The rats were adapted to the experimental environment three days before the experiments began. All animals were tested during the light cycle at the same time of day (9:00 a.m.–14:00 a.m.). All procedures were approved by the Institutional Animal Care Committee of the University of São Paulo (protocol number 26, page 84, book 02 13/04/2010). Efforts were made to minimize the number of animals used and their suffering.

#### 2.1.1. Chronic Constriction Injury (CCI)

To induce neuropathic pain, chronic constriction of the sciatic nerve was performed as previously described by Bennett and Xie in 1988 [25]. Briefly, the rats were anesthetized with halothane (Cristalia, Brazil) [25], and the common sciatic nerve was exposed at the level of the middle of the thigh by blunt dissection through the biceps femoris. The nerve was freed from adhering tissue proximal to the sciatic trifurcation (approximately 7 mm), and 4 ligatures (4.0 chromic gut) were tied loosely around the nerve at a spacing of approximately 1 mm spacing. Great care was taken to tie the ligatures such that the diameter of the nerve was only slightly constricted. The incision was closed in layers. In the sham-operated rats, the sciatic nerve was exposed, but no ligatures were applied. Each rat was closely observed during recovery from anesthesia and then returned to the home cage and carefully observed for the subsequent 24 hours. During the 5-day period after CCI, walking and cage exploration, the degree of limping, and the conditions of the hind paw, including signs of excessive grooming or autotomy, were all closely observed. We used 10 animals per group.

#### 2.1.2. Neural Mobilization Technique (NM)

The NM technique used here has been described by Butler (1991) and adapted by our laboratory [22, 24]. Briefly, rats were anesthetized with halothane with a continuous flow of medicinal oxygen throughout the procedure (5 mL/L). After anesthesia, the animals were positioned in the left lateral position to mobilize the right side (ipsilateral to the CCI). The right knee joint was then positioned in full extension (at 0 degrees) and remained in this position throughout the session. The right hip joint was bent between 70 and 80 degrees with the knee in the extended position until a small amount of resistance induced by stretching the compartimentum femoris posterior muscles (i.e., the biceps m., femoris m., semimembranosus m., and semitendinosus m.) was encountered. After the therapist felt this resistance, the angle of the joint was interrupted. At this time, the ankle joint was angled between 30 and 45 degrees using the aforementioned technique. After all joints were positioned to induce minimal resistance from the muscles, oscillatory movements were initiated. The right ankle joint was manipulated in the dorsiflexion dimension (30 to 45 degrees) at approximately 20 oscillations per minute for 2 minutes followed by a 25-second rest. The treatment required ten minutes, and, in the last minute, the cervical spine was fully flexed to tension the entire neuraxis. NM treatments were initiated 14 days after the injury or sham procedure, and NM sessions occurred every other day for a total of 10 sessions.

#### 2.1.3. Immunoblotting

Western blotting analyses were performed on samples from individual animals (*N* = 5/group). Neuropathic (CCI), sham and naive rats were sacrificed by decapitation under light isoflurane anesthesia. The thalamus and midbrain were quickly removed and homogenized in an extraction buffer containing 100 mM Tris, 10 mM EDTA, 2 mM PMSF, and 10 *μ*g/mL aprotinin at a pH of 7.4. After extraction, the homogenates were centrifuged at 11.5*g* for 20 min, and the protein concentrations of the supernatants were determined using the Bradford protein assay with albumin as the standard (Bio-Rad) [26]. Samples containing 75 *μ*g of protein were loaded on 12% acrylamide gels and electrotransferred onto nitrocellulose membranes using a Bio-Rad miniature transfer apparatus for 1.5 h at 120 V. After transfer, the membranes were treated for 2 h at room temperature with a blocking solution containing 5% powdered milk, washed, and incubated overnight at 4°C with an anti-GFAP (1 : 1000, monoclonal anti-glial fibrillary acidic protein, clone G-A-5; Sigma), a monoclonal anti-OX-42 (1 : 1000, purified mouse anti-rat Cd11b/c monoclonal antibody; BD Bioscience Pharmingen), or an anti-BDNF (AB1779SP, 1 : 1000, Chemicon International). The membranes were then washed and incubated for 2 h at room temperature with a peroxidase-conjugated, anti-rabbit secondary antibody and diluted 1 : 5000 (ZIMED Laboratories Inc.) with anti-mouse secondary antibody (GE Healthcare). *β*-Actin was used as an internal control (monoclonal mouse anti-*β*-actin 1 : 15000, Sigma). The specifically bound antibody was visualized using a chemoluminescence kit (Amersham Biosciences). The blots were analyzed densitometrically using NIH-Scion Image 4.0.2 and quantified with optical densitometry of the developed autoradiographs* (Scion Corporation, Release Beta 3b, NIH)*.

#### 2.1.4. Immunohistochemistry

The animals were deeply anesthetized with ketamine (5 mg/100 g body weight, i.p.) and xylazine (1 mg/100 g body weight, i.p.) and perfused through the left cardiac ventricle with phosphate buffered saline at 37°C and 4% paraformaldehyde in cold 0.1 M phosphate buffer (PB), pH 7.4. Brains were dissected out and postfixed for 2 h. After this period, they were kept in a cryoprotective 30% buffered sucrose solution in PB for at least 4 h until sectioning.

The brain sections (30 *μ*m) were obtained on a sliding microtome adapted for cryosectioning. The sections were incubated free-floating for 12–16 h with GFAP (monoclonal anti-glial fibrillary acidic protein, clone G-A-5; Sigma/EUA), OX-42 (purified mouse anti-rat Cd11b/c monoclonal antibody; BD Bioscience Pharmigen), and BDNF (AB1779SP, Chemicon International) all of them diluted 1 : 1000 in 0.3% of Triton X-100, containing 0.05% goat normal serum.

Following three washes of 10 min each with PB, sections were incubated for 2 h with the biotinylated secondary antibody (goat anti-mouse IgG or goat anti-rabbit IgG, Jackson ImmunoResearch, 1 : 200) and then with the avidin-biotin complex (1 : 100; ABC Elite kit, Vector Laboratories). After washing, the sections were reacted with 0.05% 3,30-diaminobenzidine and 0.01% hydrogen peroxide in PB. Intensification was conducted with 0.05% osmium tetroxide in water. The sections were mounted on gelatinized slides, dehydrated, cleared, and cover-slipped.

CCI, CCI, with NM and naive animals (used as controls) were evaluated. Controls for the immunohistochemistry experiments consisted of the omission of primary antibodies, and no staining was observed in these cases. The material was analyzed on a light microscope and digital images were collected. Figures were mounted with Adobe Photoshop CS. Manipulation of the images was restricted to threshold and brightness adjustments of the whole image.

## 3. Statistical Analyses

Statistical analyses of the data were performed with GraphPad Prism, version 4.02 (GraphPad Software Inc.). All data are expressed as the mean ± SEM. Statistical comparisons of more than 2 groups were performed with analyses of variance (ANOVAs), and differences between means were tested using Tukey's tests. In all cases, *p* < 0.05 was considered statistically significant [27].

## 4. Results

### 4.1. Effects of NM on GFAP Expression and Location

**Figure 1:**
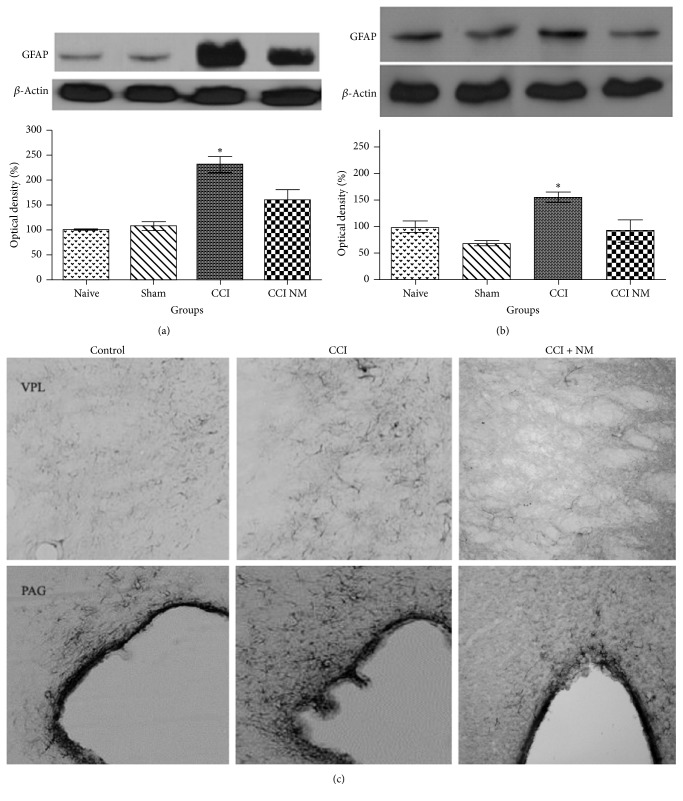
*Densitometric analyses of GFAP protein levels in thalamus (a), midbrain (b), and immunohistochemistry locating VPL and PAG with GFAP immunoreactivity (c)*. The normalized averages of the naive and experimental groups (CCI) are reported. The means of the naive animals were taken as 100%. The data are reported as means ± SEM of 5 animals per group. ^*∗*^*p* < 0.05 compared to the naive group.

Single GFAP-positive bands were observed in thalamus and midbrain extracts of all analyzed groups. Figures 1(a)–1(c) illustrate the observed increases in GFAP protein levels in the ipsilateral (with respect to the CCIs) sides of the experimental animals across all analyzed tissues and immunohistochemistry. Densitometry analyses of the thalamus and midbrains revealed increases in GFAP expression in the animals that received CCI (129% and 65%, resp.) compared to the naive animals and decreases of 70% in the thalamus and 64% in the midbrains (*p* > 0.05) of the rats that received NM treatment (CCI-NM) compared to the CCI group (Figures 1(a) and 1(b)). Immunohistochemistry showed decreased GFAP immunoreactivity in ventral posterolateral nucleus (VPL) in thalamus and periaqueductal gray (PAG) in midbrain after NM (Figure 1(c)). No significant differences in GFAP expression were observed between the sham-NM and naive groups or between the sham and sham-NM groups (data not shown).

### 4.2. Effects of NM on OX-42 Expression and Location

**Figure 2:**
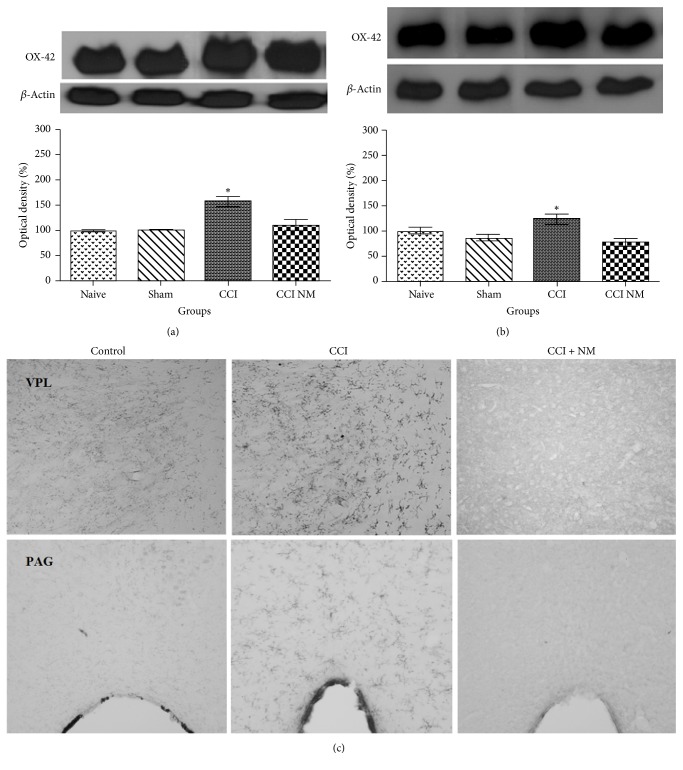
*Densitometric analyses of OX-42 protein levels in thalamus (a), midbrain (b), and immunohistochemistry locating VPL and PAG with OX-42 immunoreactivity (c)*. The normalized averages of the naive and experimental groups (CCI) are reported. The means of the naive animals were taken as 100%. The data are reported as means ± SEM of 5 animals per group. ^*∗*^*p* < 0.05 compared to the naive group.

We evaluated the expression of OX-42 protein in thalamus and midbrain tissues as described above. The results revealed increases in OX-42 levels of 58% in the thalamus and 26% in the midbrain after CCI injury when compared to naive animals. After NM treatment, we observed decreases in OX-42 expression in thalamus and midbrain of 47% and 46% (*p* > 0.05), respectively (Figures 2(a) and 2(b)). Immunohistochemistry showed decreased OX-42 immunoreactivity in ventral posterolateral nucleus (VPL) in thalamus and periaqueductal gray (PAG) in midbrain after NM (Figure 2(c)). No differences were observed between the naive and sham-NM groups or between the sham and sham-NM groups (data not shown).

### 4.3. Effects of NM on BDNF Expression and Location

**Figure 3:**
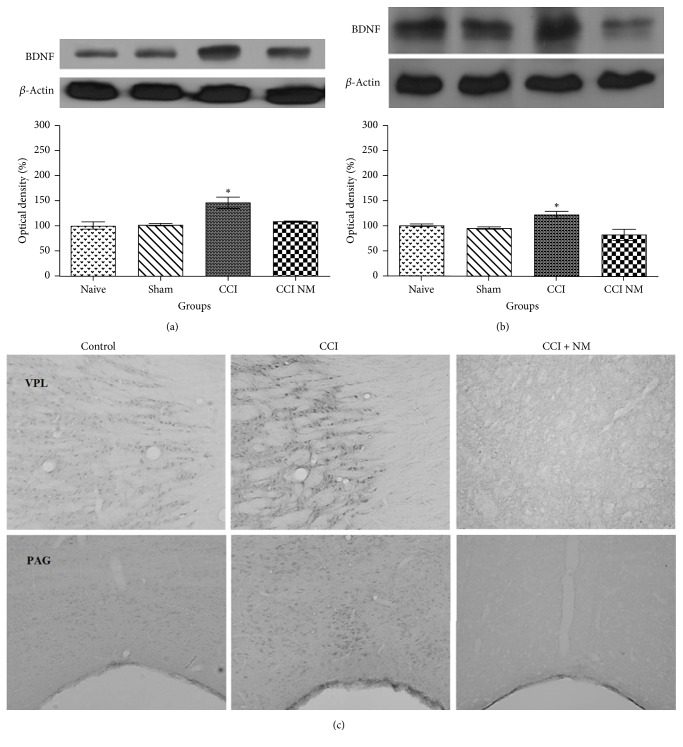
*Densitometric analyses of BDNF protein levels in thalamus (a), midbrain (b), and immunohistochemistry locating VPL and PAG with BDNF immunoreactivity (c)*. The normalized averages of the naive and experimental groups (CCI) are reported. The means for the naive animals were taken as 100%. The data are reported as means ± SEM of 5 animals per group. ^*∗*^*p* < 0.05 compared to the naive group.

We also analyzed the expressions of BDNF protein in thalamus and midbrain tissues as described above. The results revealed increases in BDNF levels of 45% in the thalamus and 27% in the midbrain (*p* > 0.05) after CCI injury compared to naive animals. Moreover, decreases in BDNF expression in thalamus and midbrain of 36% and 41% (*p* > 0.05), respectively, were observed after NM treatment (CCI-NM) (Figures 3(a) and 3(b)). Immunohistochemistry showed decreased BDNF immunoreactivity in ventral posterolateral nucleus (VPL) in thalamus and periaqueductal gray (PAG) in midbrain after NM (Figure 3(c)). No differences were observed between the naive and sham-NM groups or between the sham and sham-NM groups (data not shown).

No differences in *β*-actin levels between the control and experimental sides were observed at any tested time point (Figures 1, 2, and 3).

## 5. Discussion

Recently, our group proposed analyzing the effects of NM in rats with neuropathic pain. Previous results have shown that NM is able to decrease pain sensitivity in rats after CCI injury, which suggests that this technique could be used as an adjuvant therapy for patients with pain symptoms [22, 24]. Here, we sought to better understand the types of cells that are involved in this phenomenon. The aim of this study was to evaluate the involvements of central glial cells (GFAP and OX-42) and brain-derived neurotrophic factor (BDNF) in the thalamus and midbrain after CCI and after NM treatment in rats.

In many pathological conditions, tissue injury is the immediate cause of pain. These injuries result in local release of many chemical mediators that act on nerve endings to directly activate them or to exacerbate their sensitivities to other forms of stimulation (e.g., hyperalgesia and allodynia) [28, 29]. When used as an experimental model in rats, CCI of the sciatic nerve induces pain-related behaviors that are similar to those observed in humans; thus, this model is accepted as a model that resembles human neuropathic pain [25, 30, 31]. The number of therapeutic options for the management of neuropathic pain has increased [32, 33]; however, the responses of patients with this type of pain to the current treatments are not satisfactory. Clinically, application of the NM technique has produced excellent results [34–36]. Studies conducted by our group have standardized the technique of NM in rats with CCI and have shown that NM is effective against the painful sensitivity induced by CCI [17]. Glial-neuronal interactions have been studied in the context of enhanced nociception. Studies performed by several groups have demonstrated that microglia and astrocytes in the spinal cord are essential for the initiation and maintenance of pathological pain [3, 37–41]. In addition to glial alterations that occur in the spinal cord and nerves, studies have also observed changes in various brain regions that include thalamic microglial activation after injury to the nociceptive spinal cord [3, 42], astrocytic activation in the cingulate cortex after ligation of the sciatic nerve [43], and activation of the nucleus of the solitary tract after colon inflammation [44]. For this study, we chose to study the involvement of glial cells because of the various processes that involve these cells in models of, for example, inflammation, neuropathy, and spinal immune activation [10, 37, 38, 40, 45–47].

Our results revealed increases in glial cells (GFAP and OX-42) in the thalamus and midbrain after CCI injury relative to the control groups (i.e., the naive and sham groups). In contrast, when the animals received neural mobilization treatment, we observed the inverse result; that is, we observed decreases in GFAP (70% in the thalamus and 64% in the midbrain) and OX-42 (47% in the thalamus and 46% in the midbrain) expression compared to the CCI group that did not receive treatment; these results suggest that these cells were involved in our model. Our findings corroborate the findings of other studies showing an increase of GFAP expression after CCI injury and a decrease after administration of the N-methyl-D-aspartate (NMDA) receptor antagonist [11, 45] or treatment with low level laser therapy (Oliveira, 2017). Additionally, Raghavendra et al. (2003) showed that inhibition of microglia activity via the administration of minocycline attenuates the hypersensitivity behavior of rats in a nerve transection model of neuropathic pain [48]. Similarly, Mika et al. (2009) showed that inhibition of microglia activity via the administration of minocycline attenuates the hypersensitivity behavior of rats in a CCI model of neuropathic pain [49].

Studies have reported that, after nerve injury, neuronal metabolism is altered such that protein synthesis is increased to support the regenerative roles of neurotrophins such as nerve growth factor (NGF) and brain-derived neurotrophic factor (BDNF); both of these neurotrophins act to regulate the growth and hence survival of sensory neurons [50, 51]. Recent evidence suggests that neurotrophins, particularly BDNF, play key roles as mediators/modulators of pain [52–56]. Studies suggest that BDNF synthesis is increased not only in primary afferent neurons during the painful process but also in second-order nociceptive neurons [53, 57] and glial cells of the posterior column of the spinal cord [13, 58]. Our results revealed significant differences in the optical densities of the labeled BDNF bands. We demonstrated increases in BDNF expression after CCI injury in all tissues analyzed compared with the control group. In the animals treated with neural mobilization (NM CCI), we found decline in BDNF expression (36% in the thalamus and 41% in the midbrain) compared to the animals that did not receive treatment. These data corroborate the findings of a study by Coull et al. (2005) that demonstrated the involvement of BDNF in the generation of chronic pain and the reversal of pain after blocking the receptor for BDNF (TrkB) [13] and M'Dahoma et al. (2015) observed marked induction of microglia activation markers (OX-42, Iba1, and P-p38), proinflammatory cytokine IL-6, NMDA receptor subunit NR2B and BDNF in spinal cord, and dorsal root ganglia caused by chronic constriction injury to the sciatic nerve. A long-lasting spinal BDNF overexpression was also observed in rats injected intrathecally with BDNF, indicating an autocrine self-induction, with downstream long-lasting TrkB-mediated neuropathic-like pain. [59]. Other studies have identified increases in the synthesis of this neurotrophin in the DRGs of animals with neuropathic and inflammatory pain [60, 61]. Besides that, we could locate the GFAP, OX-42, and BDNF expression in VPL and PAG areas with the immunohistochemistry technique and it was able to show the decreased immunoreactivity in all the proteins after neural mobilization treatment.

In summary, the increased optical densities for astrocytes, microglia, and BDNF that we observed in the thalamus and midbrain after CCI injury were reversed by neural mobilization treatment. Other treatments were able to decrease the BDNF after pain conditions, as shown with the transcranial direct current stimulation technique on pain behavior and BDNF levels in ovariectomized rats [62]. And swim exercise can normalize nerve injury-induced nerve growth factor and brain-derived neurotrophic factor (BDNF) enhanced expression in the dorsal root ganglion after partial ligation of the sciatic nerve followed by a 5-week aerobic exercise program [63]. However, those studies were not able to demonstrate the glial cells and BDNF levels in brain areas after CCI and treatment. Therefore, we believe that neural mobilization can decrease symptoms of neuropathic pain, such as hyperalgesia and allodynia, and regeneration improvement, as demonstrated in previous studies by our group [17, 24], and these findings in nociceptive behavior and regeneration may be closely related to our findings that demonstrated the involvement of glial cells and BDNF in the central nervous system. Further studies are needed to more broadly understand these processes.
